# Resveratrol enhances the functionality and improves the regeneration of mesenchymal stem cell aggregates

**DOI:** 10.1038/s12276-018-0109-y

**Published:** 2018-06-27

**Authors:** Yi-Jing Wang, Pan Zhao, Bing-Dong Sui, Nu Liu, Cheng-Hu Hu, Ji Chen, Chen-Xi Zheng, An-Qi Liu, Kun Xuan, Ya-Ping Pan, Yan Jin

**Affiliations:** 1 Department of Periodontics and Oral Biology, School of Stomatology, China Medical University, Shenyang, Liaoning 110002 China; 2 General Hospital of Shenyang Military Region, Shenyang, Liaoning 110016 China; 3 State Key Laboratory of Military Stomatology & National Clinical Research Center for Oral Diseases & Shaanxi International Joint Research Center for Oral Diseases, Center for Tissue Engineering, School of Stomatology, Fourth Military Medical University, Xi’an, Shaanxi 710032 China; 4 Xi’an Institute of Tissue Engineering and Regenerative Medicine, Xi’an, Shaanxi 710032 China; 5 Department of Periodontology, Stomatological Hospital, Zunyi Medical College, Zunyi, Guizhou 563003 China

**Keywords:** Regeneration, Mesenchymal stem cells, Tissue engineering, Drug development, Translational research

## Abstract

Mesenchymal stem cell (MSC)-based regeneration, specifically cell aggregate or cell sheet engineering, is a promising approach for tissue reconstruction. Considering the advantages of ease of harvest and lack of immune rejection, the application of autologous MSCs (i.e., patients’ own MSCs) in regenerative medicine has developed considerable interest. However, the impaired cell viability and regenerative potential following MSCs impacted by disease remain a major challenge. Resveratrol (RSV) exhibits reliable and extensive rejuvenative activities that have received increasing clinical attention. Here, we uncovered that resveratrol enhances the functionality and improves the regeneration of mesenchymal stem cell aggregates. Periodontal ligament MSCs (PDLSCs) from normal control subjects (N-PDLSCs) and periodontitis patients (P-PDLSCs) were investigated. Compared to N-PDLSCs, P-PDLSCs were less capable of forming cell aggregates, and P-PDLSC aggregates showed impaired osteogenesis and regeneration. These functional declines could be mimicked in N-PDLSCs by tumor necrosis factor alpha (TNF-α) treatment. Notably, a TNF-α-induced functional decline in N-PDLSC aggregates was rescued by RSV application. More importantly, in both N-PDLSCs and P-PDLSCs, RSV promoted cell aggregate formation and improved their osteogenic potential. Furthermore, as proven ectopically in vivo, the tissue regenerative capability of P-PDLSC aggregates was also enhanced after RSV treatment during aggregate formation in vitro. Finally, in a rat in situ regeneration model, we successfully applied both N-PDLSC aggregates and P-PDLSC aggregates to repair periodontal defects upon long-term functional improvements by RSV preconditioning. Together, our data unravel a novel methodology for using pharmacology (i.e., RSV)-based cell aggregate engineering to improve the functionality and facilitate the regeneration of MSCs from both healthy and inflammatory microenvironments, shedding light on improving the application of autologous MSC-mediated regenerative medicine.

## Regenerative medicine: Red wine compound helps rejuvenate stem cells

A molecule found in the skin of red grapes enhances the regenerative capacity of adult stem cells. Researchers in China led by Yan Jin from Fourth Military Medical University, Xi’an, and Ya-Ping Pan from China Medical University, Shenyang, isolated stem cells capable of making the different specialized cells found in the skeletal tissues from the connective tissue located at the root of tooth-bearing bones. They showed that stem cells taken from people with serious gum disease could not mature and develop into tissue-forming aggregates as well as stem cells taken from healthy individuals. Treatment of healthy stem cells with a pro-inflammatory signaling molecule impaired development, but subsequent exposure to resveratrol, the compound in grape skin, restored cellular health. Resveratrol also promoted regeneration in the diseased stem cells and in rodent models of gum disease.

## Introduction

Mesenchymal stem cells (also known as mesenchymal stromal cells or MSCs) have been extensively investigated in the regenerative therapy of various injuries and diseases in clinics^1–3^. In particular, cell aggregate engineering (also termed cell sheet technology) has been developed as a promising strategy to improve MSC-mediated regeneration^4–6^. Furthermore, the application of autologous MSCs has developed considerable interest with immense potential, notably due to their advantages of easy harvesting and lack of immune rejection^1–3^. Transplantation of autologous MSCs has been widely evaluated in clinical trials for a variety of diseases, with both encouraging results and conditional efficacies^7–9^. Underlying their limiting clinical utility, MSCs from diseased conditions are recognized to show impaired regenerative capability^10–12^, especially due to the critical detrimental effects of an inflammatory microenvironment on MSC-based regeneration^11,13^. Despite our recent work identifying small-molecule compounds to counteract inflammatory insults on MSCs^14^, pharmacological solutions to promote tissue regeneration of MSCs derived from inflammatory microenvironments remain largely unestablished.

Resveratrol (RSV) is a natural phytoalexin that exhibits reliable and widespread rejuvenative effects in various animal models, tissues and organs, and most notably, in stem cells^15,16^. For MSCs, RSV has effects on cell viability, osteogenic differentiation, and paracrine secretion in vitro^17,18^. When administered in vivo, in combination with MSCs, RSV enhances MSC-mediated liver and cardiac regeneration by improving the homing and survival of MSCs^19,20^. On the other hand, RSV has been reported to have anti-inflammatory properties and inhibitory effects on the nuclear factor kappaB (NFkB) pathway, a key inflammatory signaling pathway^21–23^. Reports indicate that the application of RSV in tissue engineering modulates inflammatory responses and enhances bone formation^24,25^. Given the above findings, we hypothesize that RSV application may serve as a feasible method to promote the tissue regeneration of MSCs derived from inflammatory microenvironments.

Previously, we isolated periodontal ligament stem cells (PDLSCs) from subjects with normal periodontal condition (N-PDLSCs) and from patients with periodontitis (P-PDLSCs) and found that P-PDLSCs have impaired osteogenic differentiation^26,27^. In this current study, we further show that P-PDLSCs are less capable of forming cell aggregates and that the P-PDLSC aggregates have weaker osteogenic and regenerative potential, which could be mimicked in N-PDLSCs by treatment with the inflammatory cytokine tumor necrosis factor alpha (ΤΝF-α). Importantly, RSV application could restore cell aggregate formation and osteogenesis in both normal and TNF-α-treated N-PDLSCs and in P-PDLSC aggregates. Osteogenic and regenerative improvements of RSV on P-PDLSC aggregates were verified ectopically in vivo. Furthermore, after demonstrating functional improvements via RSV treatment for in situ regeneration, we successfully applied both N-PDLSC aggregates and P-PDLSC aggregates to repair periodontal defects. Together, our data unravel a novel methodology for using pharmacology (i.e., RSV)-based cell aggregate engineering to improve the functionality and facilitate the regeneration of MSCs derived from both healthy and inflammatory microenvironments, thereby shedding light on improving the clinical application of autologous MSC-mediated regenerative medicine.

## Materials and methods

### Isolation, culture, and verification of human PDLSCs

Human sample collection and experiments were performed according to the Declaration of Helsinki in its newest version. Human third molars were extracted from donors with informed consents at the Dental Clinic, which was pre-approved by the School of Stomatology at Fourth Military Medical University. Donors with normal periodontal conditions (25–41 years of age, *n* = 3/gender) and donors with periodontitis (without systemic conditions and odontogenic tumors) (25–41 years of age, *n* = 3/gender) were recruited. The classification of periodontitis was based on clinical examination and radiography assessment using defined variables with the presence of bleeding on probing, a probing depth >5 mm and a loss of attachment level >3 mm^26,28^.

PDL samples were collected by scraping the middle third of the root surface and put into culture media. The isolation and culturing of human PDLSCs were performed as previously described^10,26,28^. Briefly, the sampled human PDLs were digested with 3 mg/ml collagenase I (Sigma-Aldrich, USA) for 2 h at 37 °C to obtain single-cell suspensions. Cells were cultured in normal culture media containing α-minimal essential medium (α-MEM; Invitrogen, USA) supplemented with 10% fetal bovine serum (FBS, Invitrogen), 2 mM l-glutamine (Invitrogen), 100 U/ml penicillin (Invitrogen), and 100 g/mL streptomycin (Invitrogen) in a humidified atmosphere of 5% CO_2_ at 37 °C. The media were changed every 3 days. After approximately 1 week, primary cells were gathered using 0.25% trypsin (MP Biomedicals, USA) and then seeded into 96-well plates to obtain single-cell-derived colony cultures using the limiting dilution technique. After approximately 2 weeks, different colonies were gathered as first-passage PDLSCs using 0.25% trypsin (MP Biomedicals) and then passaged. Colony-expanded purified PDLSCs at the third passage were used for further experiments^28^.

Surface marker profiling of human PDLSCs was performed accordingly based on flow cytometry^10,28^. The third-passaged PDLSCs were digested and suspended in phosphate-buffered saline (PBS) supplemented with 3% FBS at 1 × 10^6^ cells/ml. In total, 2 × 10^5^ cells per tube were added with 1 μl of fluorescein isothiocyanate (FITC)-conjugated anti-human CD29 antibody, 1 μl of PE-conjugated anti-human CD31 antibody, 1 μl of PE-conjugated anti-human CD45 antibody, 1 μl of PE-conjugated anti-human CD90 antibody, and 1 μl of FITC-conjugated anti-human CD105 antibody (all from Abcam, UK). Non-immune immunoglobulin of the same isotype was used as the negative control. PDLSCs were incubated in 4 °C for 30 min in dark and then washed twice with PBS supplemented with 3% FBS. The percentages of positively stained cells were determined with a flow cytometer (FACSAria, BD, USA) equipped with FACSDiva Version 6.1.3 software (BD).

### Establishments of PDLSC aggregates

PDLSC aggregates were constructed based on previous reports^4–6^. Briefly, the third-passaged PDLSCs were seeded in six-well culture plates at a density of 1 × 10^6^ cells/well. At confluence, PDLSCs were further cultured in cell aggregate-inducing media, i.e., normal culture media supplemented with 50 μg/ml vitamin C (Sigma-Aldrich) for 10 days until the cells at the edge of the wells wrapped. For TNF-α treatment, 10 ng/ml human recombinant TNF-α (PeproTech, USA) was added into the cell aggregate-inducing media. The dose was selected according to our previous reports using TNF-α to treat PDLSCs^29^. For RSV treatment, 10 nM RSV (Life Science Products, USA) was added into the cell aggregate-inducing media. The dose was determined based on our previous documents on the rejuvenative effects of RSV on MSCs during in vitro expansion^30^. All media were changed every 3 days and used away from light.

### Osteogenic differentiation

To induce osteogenic differentiation, PDLSC aggregates were further cultured in osteogenic-inducing media containing 50 μg/ml ascorbic acid (MP Biomedicals), 2 mM β-glycerophosphate (Sigma-Aldrich), and 10 nM dexamethasone (Sigma-Aldrich)^4,28^. For TNF-α treatment^29^, 10 ng/ml human recombinant TNF-α (PeproTech) was added into the osteogenic-inducing media. For RSV treatment^30^, 10 nM RSV (Life Science Products) was added into the osteogenic-inducing media. All media were changed every 3 days and used away from light.

After induction for 7 days, alkaline phosphatase (ALP) staining was performed^28,31^. ALP activity was determined using the ALP (AKP/ALP) detection kit (BioVision, Milpitas, USA) according to the manufacturer’s instructions^26^. After induction for 14 days, alizarin red staining was performed to determine mineralization. The quantitation of the mineralized area over total area was determined with ImageJ 1.47 software (National Institutes of Health, USA). The quantification was performed based on semi-automatic plug-ins for recording and repeating the steps of an operation. The thresholds for distinguishing the quantification objectives with the background were set based on preliminary tests with several differential photographs^31^.

### Animals

Animal experiments were approved by Fourth Military Medical University and performed following the Guidelines of Intramural Animal Use and Care Committee of Fourth Military Medical University. Animal experiments were also performed following the ARRIVE guidelines and the NIH Guide for Care and Use of Laboratory Animals. Herein, 8-week-old female nude mice (weight, 16–18 g) and 8-week-old female Sprague-Dawley rats (weight, 200–220 g) (Laboratory Animal Center, Fourth Military Medical University, China) were used. Animals were randomly assigned to different experimental groups. Investigators were blinded to the sample group allocations. The animals were maintained with good ventilation and a 12-h light/dark cycle and had ad libitum access to food and water prior to being sacrificed.

### Ectopic tissue regeneration of PDLSC aggregates in nude mice

To investigate the bone regeneration and collagen bone matrix deposition of PDLSC aggregates in vivo, N-PDLSC aggregates, P-PDLSC aggregates, and RSV-treated P-PDLSC aggregates were respectively transplanted into the dorsal region of nude mice. Calcined bovine bone (CBB) was used as the scaffold^32^, and CBB transplantation was applied as the blank control. Surgical procedures were conducted as previously stated^5^ under general anesthesia. Briefly, 30 mg of CBB was wrapped in PDLSC aggregates to form cell aggregate-biomaterial constructs, which were then placed in 5-cm culture dishes and incubated in 5 ml of normal culture media at 37 °C for 1 h to allow stable adhesion. Next, the cell aggregate-biomaterial constructs were transplanted subcutaneously into the dorsal region of nude mice (*n* *=* 6 per group). The mice were sacrificed 4 weeks post-surgery.

### In situ tissue regeneration of PDLSC aggregates in a rat periodontal defect model

The periodontal inferior alveolar bone defect model in rats was established based on published methods with minor modifications^14,33^ under general anesthesia. Briefly, the skin at the surgical site was shaved and disinfected, and full thickness incision through the skin was made along the inferior border of the right mandible. The masseter muscle and periosteum covering the buccal surface of the mandible were elevated as a flap. The alveolar bone covering the roots of the mandibular first and second molars was removed using round burs with copious saline irrigation. The coronal margin of the defects was approximately 1 mm apical of the alveolar bone crest. The defects were made at approximately 2 mm × 1 mm for the application of RSV-preconditioned P-PDLSC aggregates and 2.5 mm × 1.5 mm for the application of RSV-preconditioned N-PDLSC aggregates.

After the defects were washed with abundant saline, N-PDLSC aggregates, P-PDLSC aggregates, and RSV-treated PDLSC aggregates carrying the CBB scaffolds were implanted into the defects. CBB transplantation alone was applied as the blank control. The masseter muscle was then repositioned and sutured, and the skin incision was closed to ensure healing. Pain was effectively controlled with buprenorphine analgesic post-surgery. Rats were sacrificed at 4 weeks post-surgery.

### Micro-computed tomography analysis

For evaluation of periodontal bone regeneration after the application of PDLSC aggregates, upon sacrifice, rat mandible specimens were removed, fixed overnight in 4% paraformaldehyde, and scanned using a desktop micro-CT system (eXplore Locus SP; GE Healthcare, USA) at a resolution of 8 μm, a voltage of 80 kV, and a current of 80 μA^34–36^. Periodontal bone data were obtained at regions of interest (ROIs) in the original defect area. Data were analyzed with Micview V2.1.2 software, and quantification was performed using the parameters of bone volume per tissue volume (BV/TV) and percentages of the remaining defect area over the original defect area.

### Histological analysis

Samples of PDLSC aggregates, nude mice-regenerated tissue biopsies, and rat mandible samples after micro-CT scanning were respectively harvested and fixed overnight with 4% paraformaldehyde. The regenerated tissue biopsies in nude mice and rat mandible samples were then decalcified with 10% ethylene diamine tetraacetic acid (EDTA) (pH, 7.2–7.4). The specimens were embedded in paraffin, sectioned to 5-μm thickness, and then deparaffinized. hematoxylin and eosin staining was conducted as previously described^31^, and the thickness of cell aggregates and the percentages of bone area over total area were analyzed using ImageJ 1.47 software (National Institutes of Health). For analysis of collagen deposition by cell aggregates in vitro and in vivo, Masson’s trichrome staining was performed with a commercial kit (Sigma-Aldrich) according to the manufacturer’s instructions. The collagen index was evaluated as previously reported^37,38^. The percentages of collagen bone matrix area over total area were analyzed using ImageJ 1.47 software (National Institutes of Health).

### Immunohistochemistry analysis

Immunohistochemistry was performed according to our published methods^28^. Deparaffinized sections were treated with 0.25% trypsin (MP Biomedicals) for 30 min at 37 °C for antigen retrieval, washed, and treated with 3% hydrogen peroxide for 20 min at 37 °C. Sections were blocked with 5% bovine serum albumin (BSA) (Sigma-Aldrich) in PBS for 2 h at room temperature. Sections were then stained with primary antibodies overnight at 4 °C as follows: a rabbit anti-human Periostin antibody at a concentration of 1:200 (Abcam), a mouse anti-human p-NFκB p65 antibody at a concentration of 1:200 (Santa Cruz Biotechnology, USA), a rabbit anti-human Bone sialoprotein (Bsp) antibody at a concentration of 1:100 (Abcam), and a mouse anti-human Osteopontin (Opn) antibody at a concentration of 1:200 (Santa Cruz Biotechnology). The sections were then stained by a goat anti-rabbit secondary antibody or a rabbit anti-mouse secondary antibody (Cell Signaling Technology, USA) for 30 min at room temperature at a concentration of 1:200. Subsequently, an HRP-based Dako REAL^TM^ EnVision^TM^ Detection System (Dako, Denmark) was used to detect the immunoactivity, followed by counterstaining with hematoxylin (Sigma-Aldrich). Negative control experiments were performed by omitting the primary antibodies. Quantification of the percentages of positive stained area over total area was performed using ImageJ 1.47 software (National Institutes of Health).

### Quantitative real-time polymerase chain reaction (qRT-PCR) analysis

Total RNA was collected from PDLSC aggregates after osteogenic induction for 14 days. RNA was extracted by the addition of Trizol Reagent (Takara, Tokyo, Japan), grinded under liquid nitrogen, and purified by phenol–chloroform extraction. cDNA synthesis and PCR procedures were performed as described^28,31^. The primer sequences were as follows: *h-Alp*: forward 5′-CCTTGTAGCCAGGCCCATTG-3′, reverse 5′-GGACCATTCCCACGTCTTCAC-3′; *h-Runt-related transcription factor 2* (*Runx2*): forward 5′-CACTGGCGCTGCAACAAGA-3′, reverse 5′-CATTCCGGAGCTCAGCAGAATAA-3′; *h-Osteocalcin (Ocn)*: forward 5′-CCCAGGCGCTACCTGTATCAA-3′, reverse 5′-GGTCAGCCAACTCGTCACAGTC-3′; *h-Collagen I (Col1)*: forward 5′-GCAAGGTGTTGTGCGATGA-3′, reverse 5′-TGGTCGGTGGGTGACTCTG-3′; *h-Gapdh*: forward 5′-GGTGAAGGTCGGAGTCAACGGA-3′, reverse 5′-GAGGGATCTCGCTCCTGGAAGA-3′. The relative expression level of each gene was obtained by normalizing against *Gapdh* abundance.

### Western blot analysis

Western blot was performed in PDLSC aggregates as previously described^27,39^. Lysates were prepared using Cell Lysis Buffer (Beyotime, China). Protein was extracted, loaded on sodium dodecyl sulfate polyacrylamide gels, transferred to polyvinylidene fluoride membranes (Millipore, USA), and blocked with 5% BSA (Sigma-Aldrich) in PBST (PBS with 0.1% Tween) for 2 h at room temperature. The membranes were incubated overnight at 4 °C with the following antibodies: a rabbit anti-human Runx2 antibody (Santa Cruz Biotechnology) at a concentration of 1:1000, a mouse anti-human Col1 antibody (Abcam) at a concentration of 1:1000, a rabbit anti-human Periostin antibody (Abcam) at a concentration of 1:1000, a rabbit anti-human Sirtuin 1 (Sirt1) antibody (Abcam) at a concentration of 1:1000, a rabbit anti-human peroxisome proliferator-activated receptor gamma coactivator-1 alpha (Pgc1α) antibody (Abcam) at a concentration of 1:1000, a rabbit anti-human *p*-adenosine monophosphate-activated protein kinase (Ampk) antibody (Cell Signaling Technology) at a concentration of 1:1000, a mouse anti-human p-NFκB p65 antibody (Santa Cruz Biotechnology) at a concentration of 1:1000, a rabbit anti-human p-NFκB p65 antibody (Santa Cruz Biotechnology) at a concentration of 1:1000, and a mouse anti-human Gapdh antibody (Abcam) at a concentration of 1:4000. The membranes were then incubated with peroxidase-conjugated goat anti-rabbit or rabbit anti-mouse secondary antibodies (Boster, China) at a concentration of 1:40,000 for 1 h at room temperature. The blotted bands were visualized using an enhanced chemiluminescence Kit (Amersham Biosciences, USA) and a gel imaging system (5500; Tanon, China). The gray values of the bands were analyzed using ImageJ 1.47 software (National Institutes of Health).

### Statistical analysis

All the results are presented as the mean ± standard deviation (SD). Data were analyzed using two-tailed Student’s *t-*tests (for two-group comparisons) or one-way analysis of variance (ANOVA) followed by the Newman–Keuls post hoc tests (for multiple group comparisons) with GraphPad Prism 5.01 software. *P-*values <0.05 were considered statistically significant.

## Results

### PDLSCs from patients with periodontitis have impaired cell aggregate formation and impaired cell aggregate osteogenesis

**Fig. 1:**
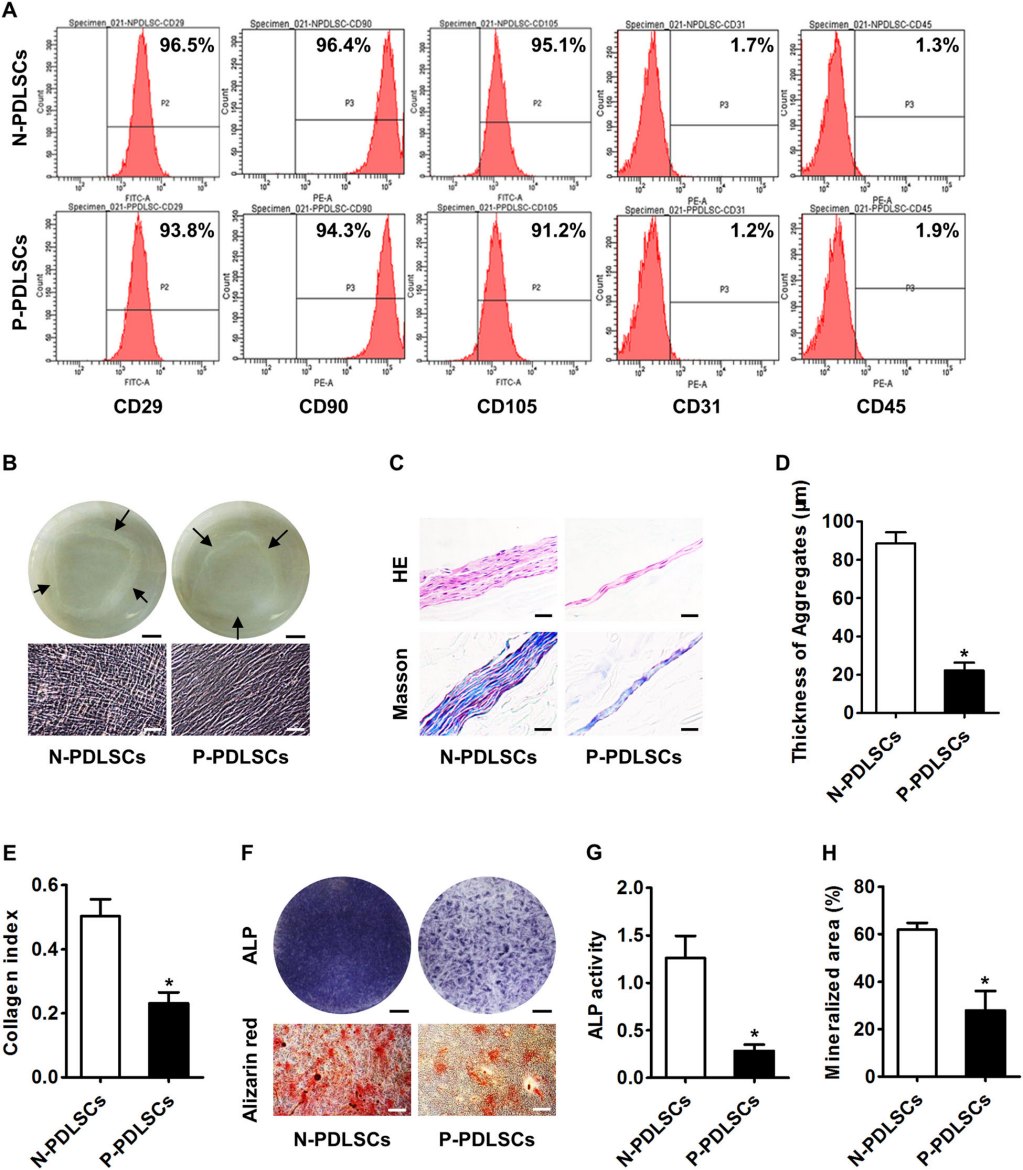
Mesenchymal stem cells (MSCs) isolated from the periodontal ligaments of patients with periodontitis showed impaired capabilities of cell aggregate formation and osteogenic differentiation. **a** Flow cytometric analysis of cell surface markers demonstrated successful isolation and culturing of periodontal ligament stem cells (PDLSCs) from subjects showing normal periodontal condition (N-PDLSCs) and patients with periodontitis (P-PDLSCs). **b** Cell aggregates formed by PDLSCs were observed photographically (top) and microscopically (bottom). Bars: 5 mm (top) and 50 μm (bottom). **c**–**e** Histological analysis of PDLSC aggregates by HE and Masson’s trichrome staining (**c**) showed that P-PDLSCs formed thinner aggregates (**d**) with less collagen deposition €. Bars: 50 μm. **f** Osteogenesis of PDLSC aggregates demonstrated by alkaline phosphatase (ALP) and alizarin red staining. Bars: 5 mm (top) and 50 μm (bottom). **g**, **h** P-PDLSC aggregates showed lower ALP activity (**g**) and weaker mineralized capability (**h**) in osteogenic differentiation. *n* = 3 per group (**a**) and *n* = 6 per group (**b**–**h**). The data represent the mean ± standard deviation (SD). *P < 0.05

To investigate whether the cell aggregates of MSCs derived from inflammatory microenvironments have impaired regenerative potential, we isolated PDLSCs from both subjects with normal periodontal conditions (N-PDLSCs) and from patients with periodontitis (P-PDLSCs). Flow cytometric analysis confirmed the successful culturing of both N-PDLSCs and P-PDLSCs by validating that the surface marker expression levels were in accordance with currently recognized standards for MSCs (Fig. 1a)^10^. Subsequently, both N-PDLSC aggregates and P-PDLSC aggregates were established, with P-PDLSC aggregates showing a sparser appearance under microscopy (Fig. 1b). Histological analysis confirmed that P-PDLSC aggregates were much thinner and showed less collagen deposition than the N-PDLSC aggregates (Fig. 1c–e). Furthermore, when induced for osteogenesis, P-PDLSC aggregates showed a lower ALP activity and a weaker mineralizing capability than N-PDLSC aggregates (Fig. 1f–h). These results suggested that MSCs derived from inflammatory microenvironments had impaired cell aggregate formation and impaired osteogenic differentiation.

### RSV preserves the aggregate formation ability and osteogenesis of N-PDLSC aggregates during inflammatory cytokine TNF-α treatment

**Fig. 2:**
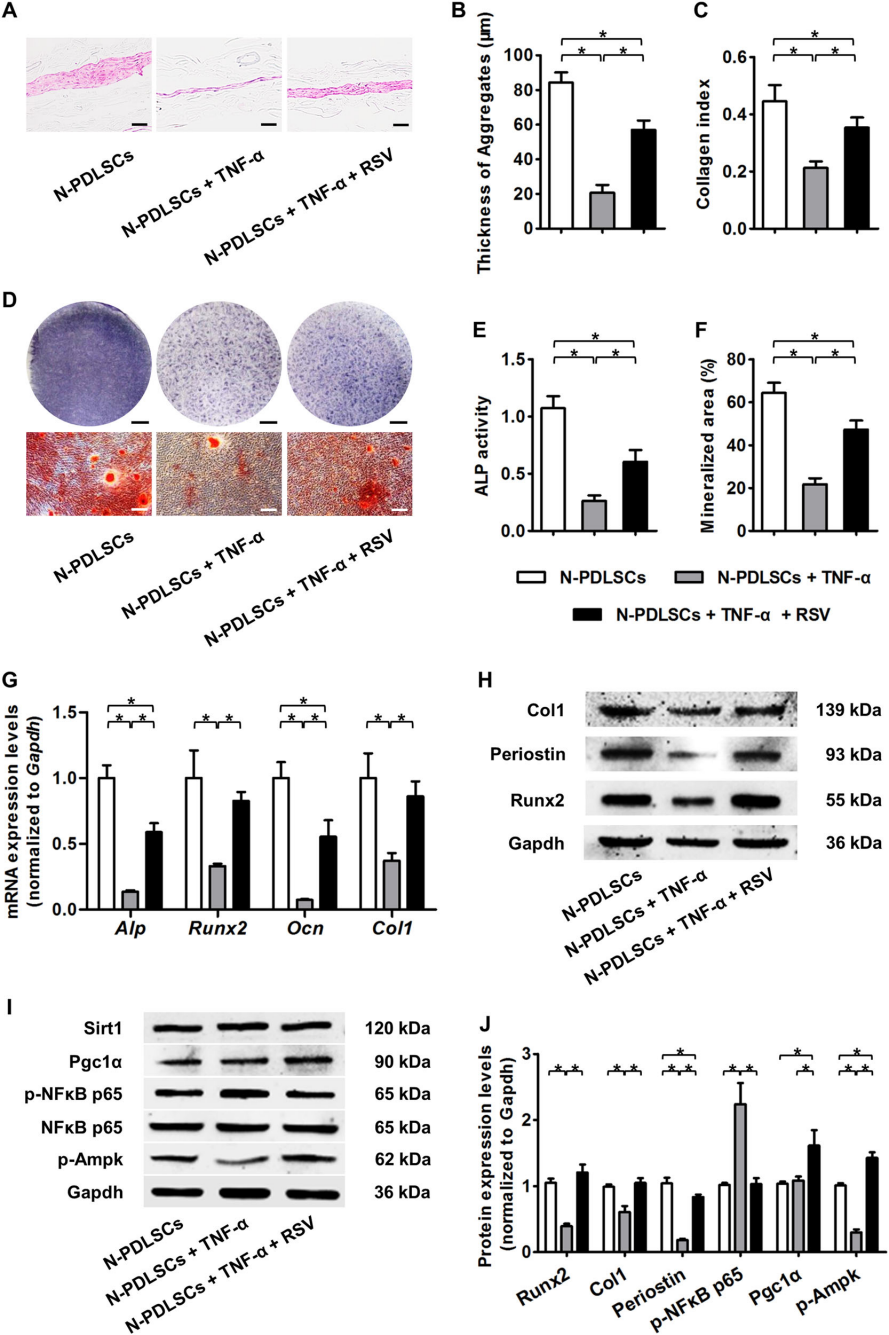
Resveratrol (RSV) rescued the cell aggregate formation and osteogenic differentiation of N-PDLSCs under inflammatory cytokine tumor necrosis factor alpha (TNF-α) treatment. **a**–**c** Histological analysis of PDLSC aggregates (**a**) showed that N-PDLSCs under TNF-α treatment formed thinner aggregates (**b**) with less collagen deposition (**c**), which could be rescued by RSV application. Bars: 50 μm. **d** Osteogenesis of PDLSC aggregates demonstrated by ALP (top) and alizarin red staining (bottom). Bars: 5 mm (top) and 50 μm (bottom). **e**, **f** RSV treatment restored the ALP activity (**e**) and mineralizing activity (**f**) of N-PDLSC aggregates under TNF-α application. **g** Quantitative real-time polymerase chain reaction (qRT-PCR) analysis of the osteogenic marker genes *Alp*, *Runt-related transcription factor 2* (*Runx2*), *Osteocalcin* (*Ocn*), and *Collagen I* (*Col1*) in PDLSC aggregates after osteogenic induction. The data showed that RSV rescued the osteogenic potential of N-PDLSC aggregates under TNF-α treatment. **h** Western blot analysis of the extracellular matrix (ECM) and osteogenic marker genes Col1, Periostin, and Runx2 in PDLSC aggregates. **i** Western blot analysis of molecular targets related to key signaling pathways in PDLSC aggregates: the Sirtuin 1 (Sirt1)-peroxisome proliferator-activated receptor gamma coactivator-1 alpha (Pgc1α) pathway; the nuclear factor kappaB (NFκB) pathway; and the adenosine monophosphate-activated protein kinase (AMPK) pathway. **j** Western blot analysis showed that RSV improved osteogenesis and ECM deposition in N-PDLSC aggregates under TNF-α treatment, possibly by inhibiting the NFκB pathway and stimulating the Pgc1α and AMPK pathways. TNF-α was applied at 10 ng/ml, and RSV was applied at 10 nM in all the experiments. *n* = 6 per group (**a**–**f**) and *n* = 3 per group (**g**–**j**). The data represent the mean ± SD. *P < 0.05

To confirm that the P-PDLSC cell aggregate formation and osteogenesis impairments were indeed attributed to inflammation, we treated N-PDLSCs with the inflammatory cytokine TNF-α, thus mimicking inflammatory microenvironments in vitro. The data showed that TNF-α treatment substantially inhibited the cell aggregate formation of N-PDLSCs (Fig. 2a–c) and suppressed the osteogenesis of N-PDLSC aggregates (Fig. 2d–f), and a corresponding downregulation of both the mRNA and protein expression levels of the osteogenic and extracellular matrix (ECM) marker genes *Alp*, *Runx2*, *Ocn*, *Col1*, and *Periostin* was observed (Fig. 2g, h).

Next, we examined whether RSV could maintain proper cell aggregate formation and osteogenic differentiation of TNF-α-treated N-PDLSCs. Histological analysis demonstrated that N-PDLSCs treated with both RSV and TNF-α formed thicker aggregates with more collagen deposition than simple TNF-α-treated N-PDLSCs (Fig. 2a–c). Furthermore, when induced for osteogenesis, RSV-treated N-PDLSC aggregates were more resistant to the inhibitory effects of TNF-α on ALP activity and mineralizing capability (Fig. 2d–f). These changes were confirmed at both the mRNA and the protein level, indicating that RSV application could reverse the suppressive impacts of TNF-α on the expression of osteogenic and ECM marker genes in N-PDLSC aggregates (Fig. 2g, h).

To dissect the molecular mechanisms underlying the protective effects of RSV on N-PDLSCs against inflammation, we investigated the changes in the key inflammatory pathway NFκB signaling^11,26^ and the reported downstream mediators of RSV effects Sirt1-Pgc1α signaling and AMPK signaling^15,40^. Western blot analysis in N-PDLSC aggregates demonstrated that p-NFκB p65 expression was upregulated by TNF-α, which could be significantly restored by RSV, without affecting the total NFκB p65 expression. Furthermore, RSV application rescued p-Ampk levels in TNF-α-treated N-PDLSC aggregates and promoted Pgc1α expression without modulating Sirt1 (Fig. 2i, j). The above data collectively suggested that RSV preserves cell aggregate formation and the osteogenic differentiation of N-PDLSCs under inflammatory cytokine TNF-α treatment, which might be molecularly attributed to the RSV restoration of NFκB signaling and stimulations of the Sirt1-Pgc1α and AMPK pathways.

### RSV treatment improves the osteogenic potential and formation of both N-PDLSC and P-PDLSC aggregates

**Fig. 3:**
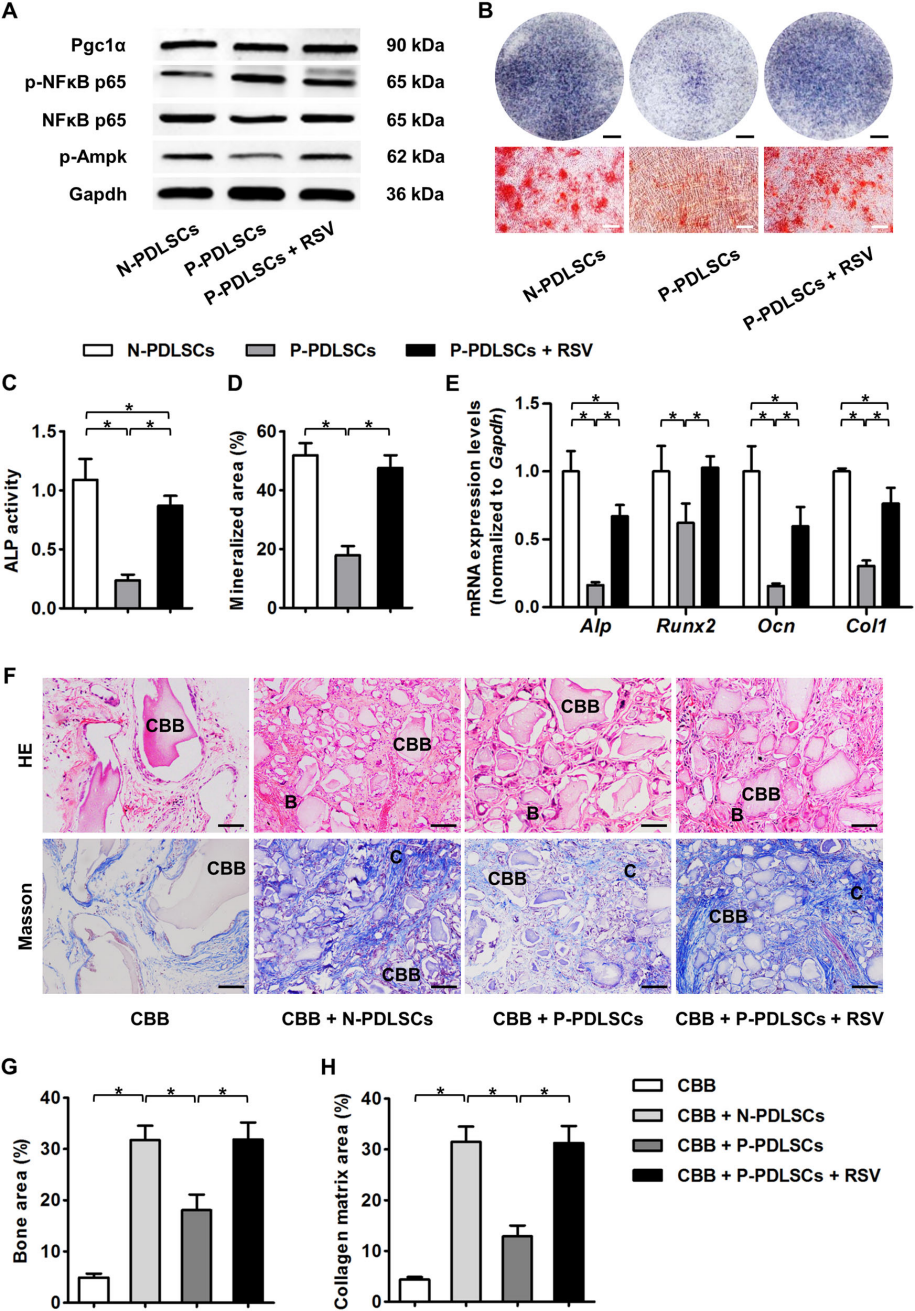
RSV treatment improved the osteogenic and bone regenerative potential of P-PDLSCs. **a** Western blot analysis of molecular targets demonstrated inhibition of the NFκB pathway and stimulation of the AMPK pathway by RSV in P-PDLSC aggregates. **b** Osteogenesis of PDLSC aggregates demonstrated by ALP (top) and alizarin red staining (bottom). Bars: 5 mm (top) and 50 μm (bottom). **c**, **d** RSV treatment improved the ALP activity (**c**) and mineralized capability (**d**) of P-PDLSC aggregates. **e** qRT-PCR analysis of the osteogenic marker genes *Alp*, *Runx2*, *Ocn*, and *Col1* in PDLSC aggregates after osteogenic induction. The data showed that RSV promoted the osteogenic potential of P-PDLSC aggregates. **f**–**h** Histological analysis of ectopic tissue regeneration of PDLSC aggregates in nude mice by HE and Masson’s trichrome staining (**f**) showed that P-PDLSC aggregates regenerated less bone (denoted by B) (**g**) and formed less collagen bone matrix (denoted by C) (**h**), which could be promoted by RSV treatment. Calcined bovine bone (CBB) was used as the scaffold. RSV was applied at 10 nM throughout the in vitro treatments. Bars: 50 μm. *n* = 3 per group (**a**) and *n* = 6 per group (**b**–**h**). The data represent the mean ± SD. *P < 0.05

Next, we verified the positive effects of RSV on P-PDLSC aggregates. We confirmed that RSV treatment also inhibited NFκB signaling in P-PDLSC aggregates, observing upregulation of only p-Ampk and not Pgc1α expression (Fig. 3a). These molecular changes were correlated with an improved osteogenic potential of P-PDLSC aggregates under RSV treatment, as shown by the RSV promotion of ALP activity, mineralization, and expression levels of osteogenic marker genes in P-PDLSC aggregates (Fig. 3b–e). Furthermore, when RSV-treated P-PDLSC aggregates were implanted in the dorsal region of nude mice, more bone tissue and collagen bone matrix could be regenerated compared with that achieved with P-PDLSC aggregates not treated with RSV. Importantly, the regenerative potential of RSV-treated P-PDLSC aggregates was even comparable to that of N-PDLSC aggregates (Fig. 3f–h). These data suggested that RSV treatment substantially improved the osteogenic and regenerative potential of P-PDLSC aggregates.

**Fig. 4:**
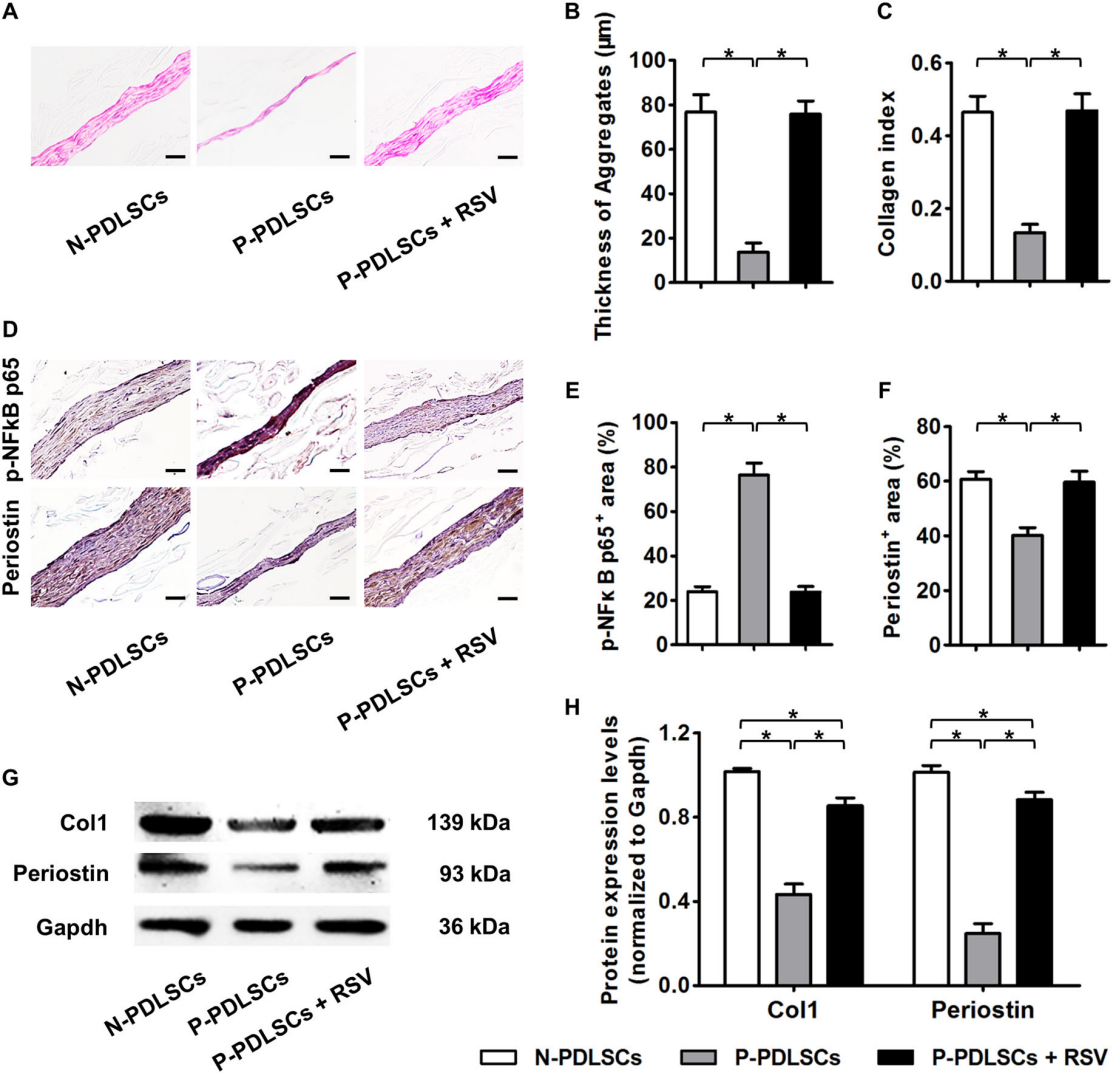
RSV treatment promoted the cell aggregate formation of P-PDLSCs. **a**–**c** Histological analysis of PDLSC aggregates (**a**) showed that RSV treatment promoted the cell aggregate formation (**b**) and collagen deposition (**c**) of P-PDLSCs. Bars: 50 μm. **d–f** Immunohistochemistry analysis of the key molecule of the NFκB pathway, p-NFκB p65, and the ECM marker gene Periostin in PDLSC aggregates (**d**). The data showed that RSV treatment suppressed the NFκB pathway (**e**) and improved ECM formation (**f**) in P-PDLSC aggregates. Bars: 50 μm. **g** Western blot analysis of the ECM marker genes Col1 and Periostin in PDLSC aggregates. **h** Western blot analysis showed that RSV improved ECM deposition in P-PDLSC aggregates. RSV was applied at 10 nM throughout the experiments. *n* = 6 per group (**a**–**f**) and *n* = 3 per group (**g**, **h**). The data represent the mean ± SD. *P < 0.05

We next examined whether RSV treatment also promoted proper the cell aggregate formation of P-PDLSCs. Histological analysis illustrated that RSV-treated P-PDLSCs showed improved aggregate formation capabilities and improved collagen deposition compared to those of N-PDLSCs (Fig. 4a–c). As demonstrated by immunohistochemistry analysis, the ameliorative effects of RSV were due to the restoration of p-NFkB p65 expression, which may have led to the rescue of Periostin expression in P-PDLSC aggregates (Fig. 4d–f). Western blot analysis confirmed that the expression of ECM marker genes *Col1* and *Periostin* in P-PDLSC aggregates increased after RSV treatment (Fig. 4g, h), indicating an improved formation of P-PDLSC aggregates by RSV application.

For a translational perspective, we sought to investigate the effects of RSV on N-PDLSC aggregates. As expected, the addition of RSV in the aggregate-inducing media significantly enhanced the formation of N-PDLSC aggregates (Figure S1A), encouraging thicker films (Figure S1B) and more collagen deposition (Figure S1C). Furthermore, RSV treatment improved the osteogenesis of N-PDLSC aggregates despite that the N-PDLSC aggregates already showed sufficient ALP activity and mineralization capability (Figure S1D–F). Collectively, these data suggested that RSV treatments serve as a beneficial ingredient for the regenerative application of PDLSC aggregates.

### RSV treatment enhances the periodontal regeneration and osteogenesis of PDLSC aggregates in situ

**Fig. 5:**
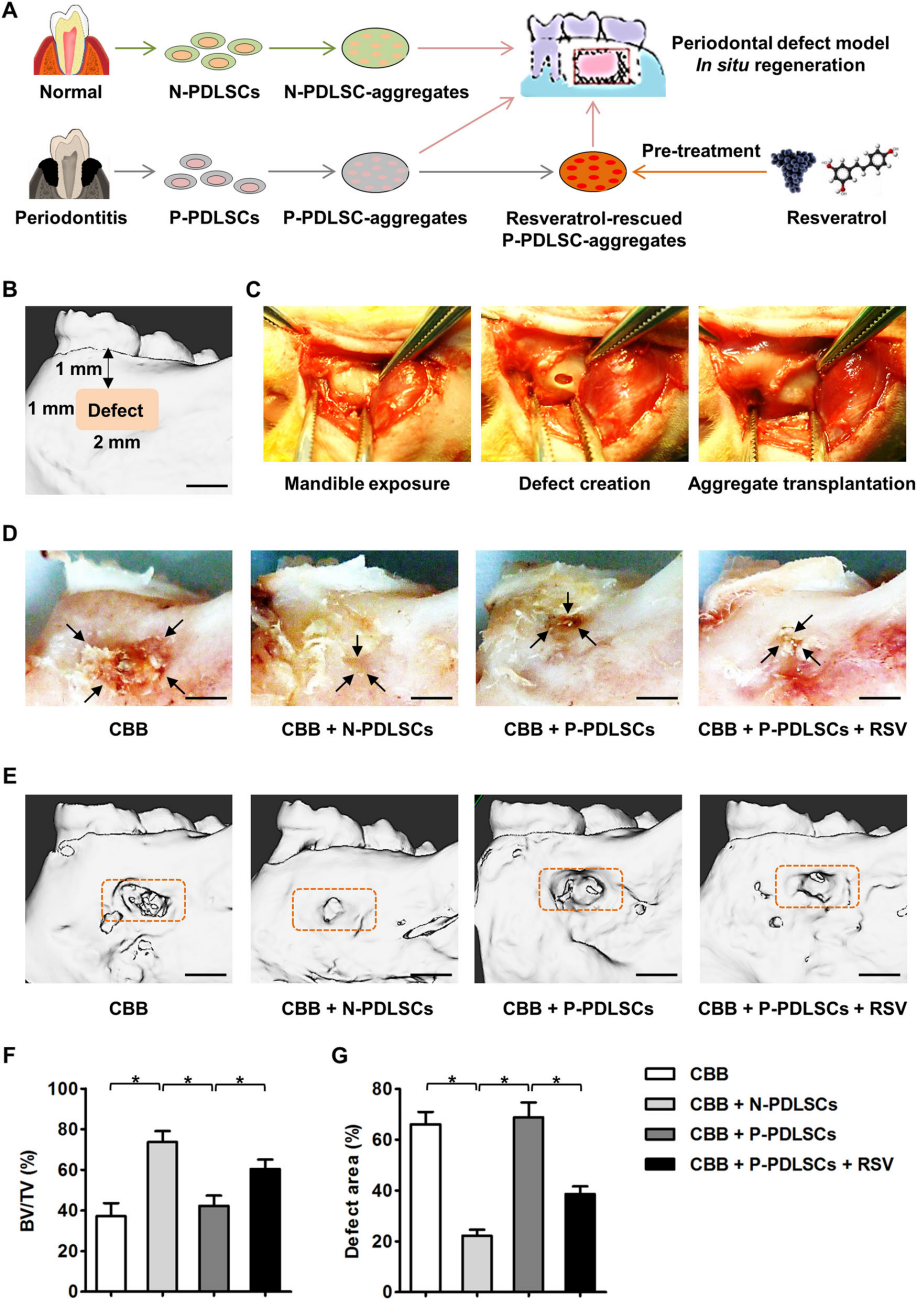
RSV treatment facilitated the alveolar bone regeneration of P-PDLSC aggregates in a rat periodontal defect model. **a** Schematic illustrating the experimental design. **b** Micro-computed tomography (micro-CT)-based schematic demonstrating the establishments of periodontal defects in the rat mandible. The defects were made approximately 1 mm beneath the alveolar bone ridge with an approximately 2 mm × 1 mm range. **c** Surgical procedures of mandible exposure (left), defect creation (middle), and aggregate transplantation (right) Bar: 1 mm. **d** Photographs of periodontal defects after PDLSC aggregate-based regeneration. CBB was used as the scaffold. Bars: 1 mm. **e**–**g** Representative micro-CT figures of periodontal defects after PDLSC aggregate-based regeneration (**e**), statistical analysis of bone volume per tissue volume (BV/TV) (**f**), and the remaining defect area over original area (**g**). The orange brackets indicate the original defects that were selected as regions of interest in statistical analysis. The data demonstrated that P-PDLSC aggregates had impaired periodontal bone regenerative capability, which could be promoted by RSV treatment. Bars: 1 mm. RSV was applied at 10 nM throughout the in vitro treatments. *n* = 6 per group. The data represent the mean ± SD. *P < 0.05

The above results prompted us to evaluate the potential of using RSV to promote the regeneration of both N-PDLSC and P-PDLSC aggregates in vivo (Fig. 5a; Figure S2A). According to previous reports, we established a periodontal defect model in rats for the in situ transplantation of PDLSC aggregates^33^ (Fig. 5a–c; Figure S2A). Four weeks post-transplantation, the N-PDLSC aggregate group showed substantially smaller periodontal defects than the scaffold alone group, while larger defects could still be observed in the P-PDLSC aggregate group. However, RSV-treated P-PDLSC aggregates demonstrated an improved capability for repairing periodontal defects (Fig. 5d). Further quantitative analysis by micro-CT confirmed the impaired regenerative potential of P-PDLSC aggregates and the ameliorative effects of RSV on P-PDLSC aggregates (Fig. 5e–g). More importantly, in a larger defect area, RSV preconditioning also improved the regenerative outcomes of the N-PDLSC aggregates (Figure S2B–E).

**Fig. 6:**
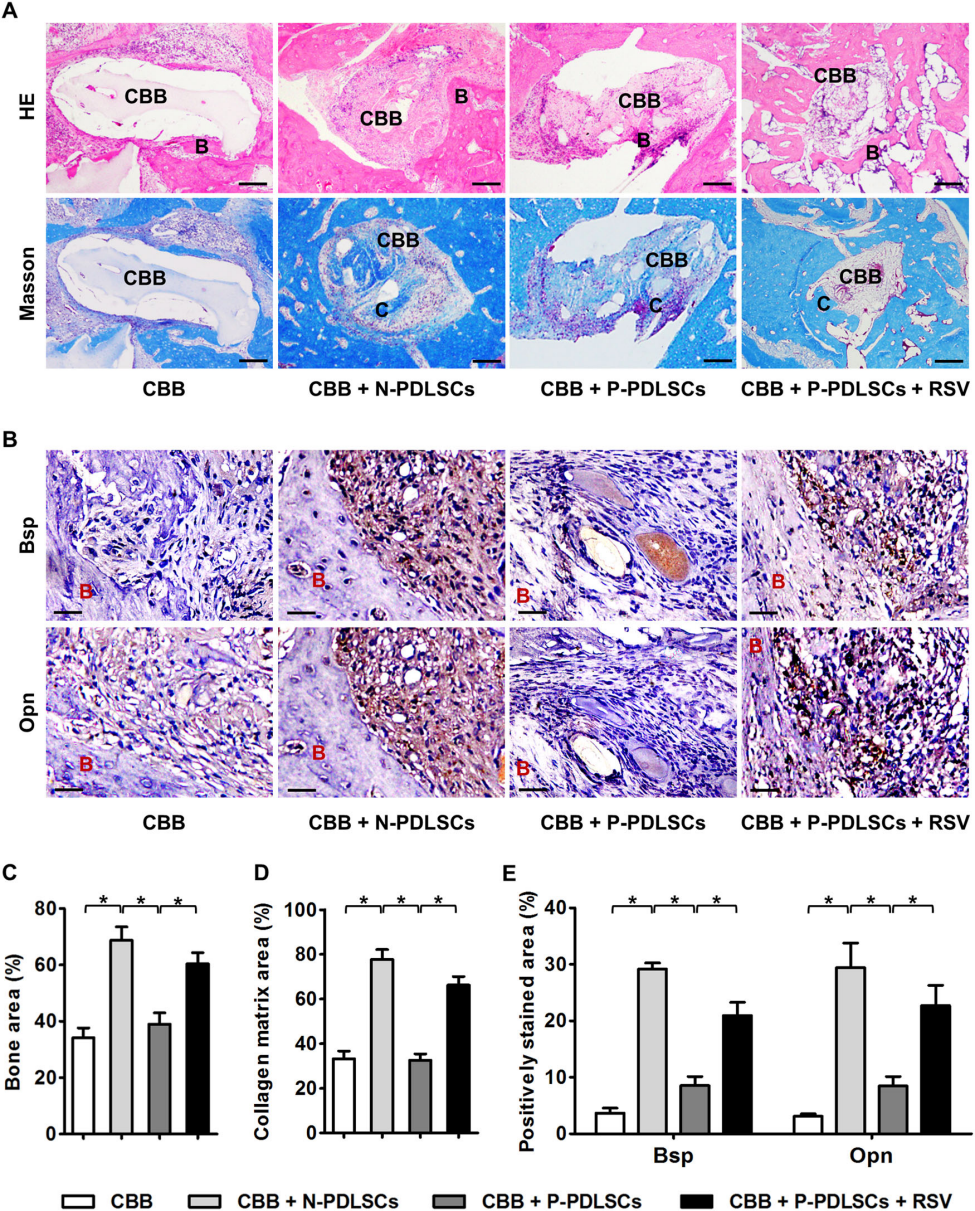
RSV treatment enhanced the periodontal regeneration and osteogenesis of P-PDLSC aggregates in vivo. **a** Histological analysis of periodontal bone regeneration (denoted by B) and collagen bone matrix deposition (denoted by C) of PDLSC aggregates by HE (top) and Masson’s trichrome staining (bottom). **b** Immunohistochemistry analysis of the osteogenic marker genes bone sialoprotein (Bsp) and osteopontin (Opn). **c**–**e** Statistical analysis showed that P-PDLSC aggregates regenerated less bone and produced less collagen matrix in periodontal defects and demonstrated weaker osteogenic potential, which could be improved by RSV treatment. RSV was applied at 10 nM throughout the in vitro treatments. *n* = 6 per group. The data represent the mean ± SD. *P < 0.05

Finally, using an in vivo model, we aimed to clarify whether the regenerative improvements of RSV on PDLSC aggregates, specifically P-PDLSC aggregates, were due to increases in osteogenesis. Histological analysis confirmed that RSV rescued both the bone tissue regeneration and collagen bone matrix deposition of P-PDLSC aggregates in situ (Fig. 6a). In the regenerative area, immunohistochemistry analysis illustrated that the bone formation marker genes *Bsp* and *Opn* were decreased in the P-PDLSC aggregate group compared with that in the N-PDLSC aggregate group, while RSV application significantly upregulated *Bsp* and *Opn* expression in tissues regenerated by P-PDLSC aggregates (Fig. 6b). Statistical analysis confirmed that the regenerative improvements of RSV on P-PDLSC aggregates were due to increases in osteogenesis (Fig. 6c–e). Together, these in vitro and in vivo data indicated that RSV treatment improved the cell aggregate formation and tissue regeneration of MSCs derived from both healthy and inflammatory microenvironments.

## Discussion

MSC-based regeneration is a promising approach for tissue reconstruction^1,2^, but the impaired regenerative potential of MSCs derived from diseased conditions, particularly inflammatory microenvironments, constitutes a major barrier to the clinical utility of autologous MSCs^10,11,13^. RSV exhibits reliable and extensive rejuvenative^15,16^ and anti-inflammatory^21–23^ activities that have attracted increasing clinical attention. In this study, we established a novel methodology for using pharmacology (i.e., RSV)-based cell aggregate engineering to facilitate the tissue regeneration of MSCs derived from both healthy and inflammatory microenvironments. We discovered that compared with N-PDLSCs, P-PDLSCs were less capable of forming cell aggregates, and the P-PDLSC aggregates had impaired osteogenic and regenerative potentials. Nevertheless, RSV treatment restored the cell aggregate formation of P-PDLSCs and osteogenesis of P-PDLSC aggregates, and more importantly, RSV applied ectopically improved the regeneration of P-PDLSC aggregates in vivo and in situ to repair periodontal bone defects. RSV preconditioning also further enhanced the functionality and regenerative performance of N-PDLSC aggregates. Our findings suggested RSV-based cell aggregate engineering as an optimal solution to enhance the functionality and improve the tissue regeneration of MSCs derived from both healthy and inflammatory microenvironments, shedding light on improving clinical the application of autologous MSC-mediated regenerative medicine.

Considering the advantages of ease to harvest and lack of immune rejection, the application of autologous MSCs (i.e., patients’ own MSCs) in regenerative medicine has developed considerable interest^1,2^. Furthermore, the transplantation of autologous MSCs is considered safe and has been extensively evaluated in clinical trials for cardiovascular, neurological, and immunological diseases with encouraging results^8,41^. However, the damaged cell viability and regenerative potential following MSCs impacted by disease, particularly inflammatory insults, remains a major limitation^10,11,13^. We attempted to use autologous PDLSC aggregates to repair intrabony defects in patients with periodontitis, but the outcomes were unsatisfying due to the strong recipient inflammation^2^. Indeed, the host inflammatory microenvironments have been well-documented to govern tissue regeneration of not only transplanted MSCs but also resident MSCs^11,13^. Liu et al.^13^ reported that recipient TNF-α and interferon-gamma (IFN-γ) impaired the regenerative potential of exogenous MSCs by inducing apoptosis and inhibiting osteogenesis. Furthermore, for endogenous MSCs, fundamental suppressive effects of inflammatory cytokines on osteogenesis have been revealed in our previous studies, underlying postmenopausal bone degeneration^11^ and periodontitis bone defects^26,27,42,43^. These diseased MSCs derived from inflammatory microenvironments were also less capable of regenerating bone when transplanted into immunocompromised mice^43^. Nevertheless, despite the various molecular mediators of inflammatory impact in MSCs being uncovered, e.g., the NFκB pathway^11,13,26,27^, pharmacological solutions to promote the tissue regeneration of MSCs derived from inflammatory microenvironments remain largely unknown. Recently, we established that a small-molecule compound derived from a Chinese herb, the osthole, improves the function of periodontitis PDLSCs via epigenetic modification^14^. In this study, we further uncovered RSV-based cell sheet engineering as an optimal solution to preserve the regeneration of MSCs from both healthy and inflammatory microenvironments, shedding light on a better clinical utility of autologous MSCs.

RSV is a natural polyphenol phytoalexin existing in red wine and grapes that was recently shown to retard aging and alleviate age-related pathological changes in various experimental models^15,16^. These rejuvenative effects might be attributed to RSV’s protection of stem cell viability, which is of vital significance for tissue homeostasis and regeneration. For MSCs, RSV has been reported to improve cell viability, osteogenesis and paracrine secretion in vitro^17,18^, and RSV enhances MSC-mediated regeneration in vivo upon co-administration^19,20^. RSV has also been reported to have anti-inflammatory properties^21–23^, thus enhancing bone formation in tissue engineering^24,25^. Here, we further demonstrated that RSV pretreatment of MSCs in culture could promote PDLSC aggregate formation against inflammation. It should be noted that both cell proliferation and the aggregation ability contribute to MSC aggregate formation and, more importantly, they should coordinate to enable the generated cells to embed within newly formed ECM. While these processes are instructive for evaluations in future works, it is of particular importance in this study that RSV preconditioning facilitates the tissue regeneration of both N-PDLSC and P-PDLSC aggregates after transplantation without further RSV administration in vivo. The methodology of RSV preconditioning in cell culture is significant (avoids the potential side effects of RSV application on off-targets) considering the widespread influences of RSV on a variety of tissues and organs despite that no detrimental impacts of RSV have been observed under any conditions^15,16^. As far as we know, this is also the first report that RSV is applied in combination with cell aggregate engineering, thus facilitating both healthy and inflammatory MSC aggregates to repair periodontal defects.

At the molecular level, the effects of RSV are exerted via stimulation of both the Sirt1 and AMPK pathways, leading to activation of the metabolic regulator Pgc1α^15,16,40^. Specifically, in MSCs, the Sirt1 pathway has been reported to be a downstream mediator of RSV effects to promote osteogenic differentiation targeting *Runx2* (refs. ^17,18^). Additionally, RSV’s impact on MSCs could also be mediated by Wnt signaling^44^, which participates in the regulation of MSC osteogenesis under inflammatory microenvironments according to our findings^26,27,42,43^. In the present study, we screened and confirmed that while the Sirt1-Pgc1α pathway may mediate RSV modulation on PDLSC aggregate formation and osteogenesis, the AMPK and NFκB pathways might be the key mediators of RSV rescue of P-PDLSC function. AMPK signaling, which senses decreases in energy charge, is known to regulate MSC differentiation^45^. NFκB signaling, the master regulator of inflammatory impact, has also been reported to be inhibited by RSV^21–23^. Reports indicate that RSV could suppress TNF-α-induced phosphorylation, NFκB p65 subunit nuclear translocation, and IκB kinase activity in addition to suppressing NFκB-dependent reporter gene transcription in a dose- and time-dependent manner^22,23^. Crosstalk among these signaling pathways may also exist (e.g., AMPK inhibition could attenuate TNF-α-induced NFκB activation^46^), but the detailed mechanisms remain to be elucidated. In addition, any potential new mechanisms related to RSV-enhanced cell aggregate formation and regeneration need to be addressed in future studies.

Cell aggregate engineering has been recognized as a promising concept for cell delivery that allows for a sheet of interconnected cells to be transplanted while also making it easier to detach the cells from the culture substrate so that their natural adhesion molecules on the cell surface and cell–cell interactions remain intact^4,5^. This technology could preserve the deposited ECM, thus mimicking cellular microenvironments in terms of various mechanical, chemical, and biological properties, which may be beneficial for cell transplantation^4,5^. Previous methods have established the induction of MSC aggregates using vitamin C rather than special equipment and have repaired large defects in bone and skin based on MSC aggregates^4–6,32^. Furthermore, we have identified several small molecules that promote the bone regenerative potential of MSC aggregates, including licochalcone A^4^ and osthole^14^. Compared with previously reported pharmacological agents, RSV has notable advantages in that it exhibits well-proven rejuvenative effects^15,16,40^ and anti-inflammatory protection^22,23^ and provides durable pre-treated influences after the transplantation of MSC aggregates. Therefore, our findings, in conjunction with other published results, highlight RSV-based cell aggregate engineering as an optimal solution to improve the tissue regeneration of MSCs derived from both healthy and inflammatory microenvironments.

In conclusion, our data unravel a novel methodology that reveals pharmacology (i.e., RSV)-based cell aggregate engineering as a solution for improving the functionality and facilitating the regeneration of MSCs derived from both healthy and inflammatory microenvironments, shedding light on improving the clinical application of autologous MSC-mediated regenerative medicine.

## Electronic supplementary material

Supplementary Information
