# Supplementary material for: Resveratrol enhances the functionality and improves the regeneration of mesenchymal stem cell aggregates
Source: Exp Mol Med. 2018 Jun 27;50(6):1–15. doi: 10.1038/s12276-018-0109-y (PMC6026147; doi:10.1038/s12276-018-0109-y)
Supplement: Supplementary file 1 — Supplementary Information [file 12276_2018_109_MOESM1_ESM.pdf]

Supplementary information

Resveratrol enhances the functionality and improves the regeneration of mesenchymal stem cell aggregates

Yi-Jing Wang [1, 2, †], Pan Zhao [3, 4, †], Bing-Dong Sui [3, †], Nu Liu [3, 5], Cheng-Hu Hu [3, 4], Ji Chen [3], Chen-Xi Zheng [3], An-Qi Liu [3], Kun Xuan [3], Ya-Ping Pan [1, *], and Yan Jin [3, *]

[1] Department of Periodontics and Oral Biology, School of Stomatology, China Medical University, Shenyang, Liaoning 110002, China.

[2] General Hospital of Shenyang Military Region, Shenyang, Liaoning 110016, China.

[3] State Key Laboratory of Military Stomatology & National Clinical Research Center for Oral Diseases & Shaanxi International Joint Research Center for Oral Diseases, Center for Tissue Engineering, School of Stomatology, Fourth Military Medical University, Xi'an, Shaanxi 710032, China.

[4] Xi'an Institute of Tissue Engineering and Regenerative Medicine, Xi'an, Shaanxi 710032, China.

[5] Department of Periodontology, Stomatological Hospital, Zunyi Medical College, Zunyi, Guizhou 563003, China.

[†] Equal contribution; [*] Corresponding authors.

Journal: Experimental & Molecular Medicine.

Supplementary information: 2 figures with their legends.

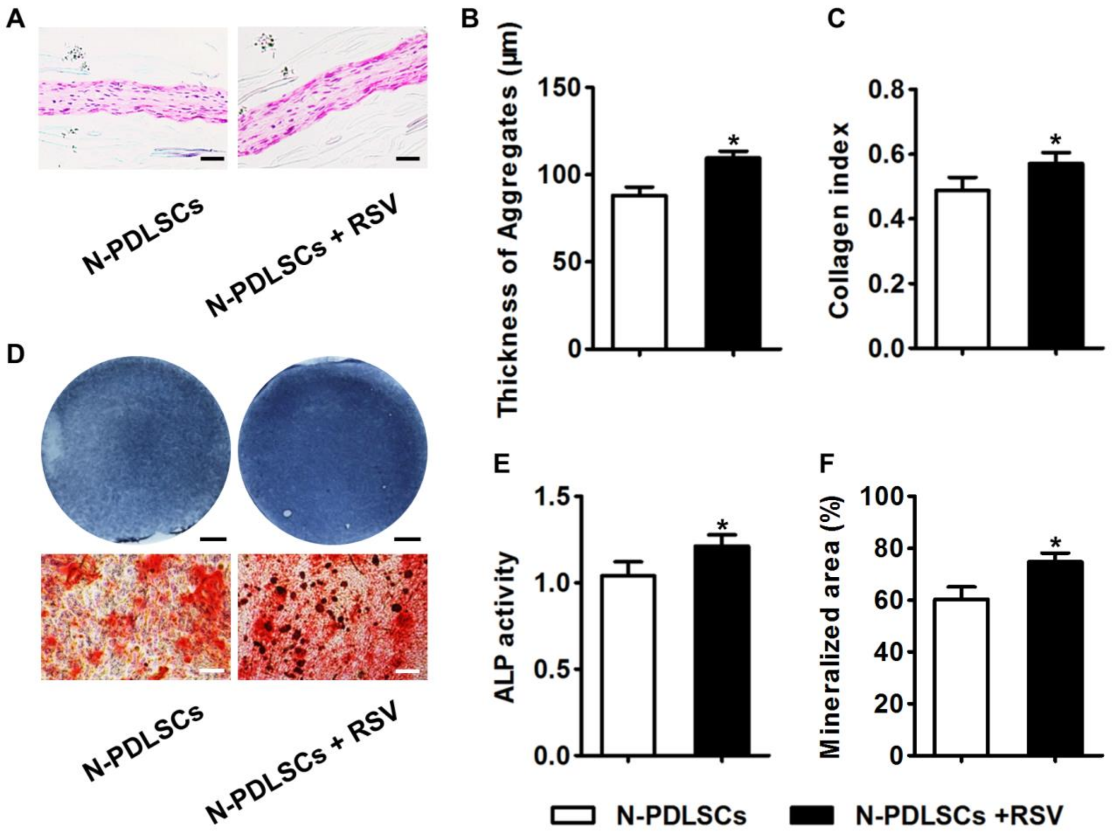

Figure S1. Resveratrol (RSV) treatment improved N-PDLSC-aggregate formation and osteogenic potential. (A-C) Histological analysis of PDLSC-aggregates (A) showed that N-PDLSCs formed thicker aggregates (B) with more collagen deposition (C) under RSV treatment. Bars: 50 μm. (D) Osteogenesis of N-PDLSC-aggregates demonstrated by alkaline phosphatase (ALP) and alizarin red staining. Bars: 5 mm (up) and 50 μm (down). (E, F) N-PDLSC-aggregates showed higher ALP activity (E) and stronger mineralized capability (F) in osteogenic differentiation under RSV treatment. RSV was applied at 10 nM throughout the *in vitro* treatments. $n = 3$ per group. Data represents mean ± standard deviation (SD). $^*P < 0.05$.

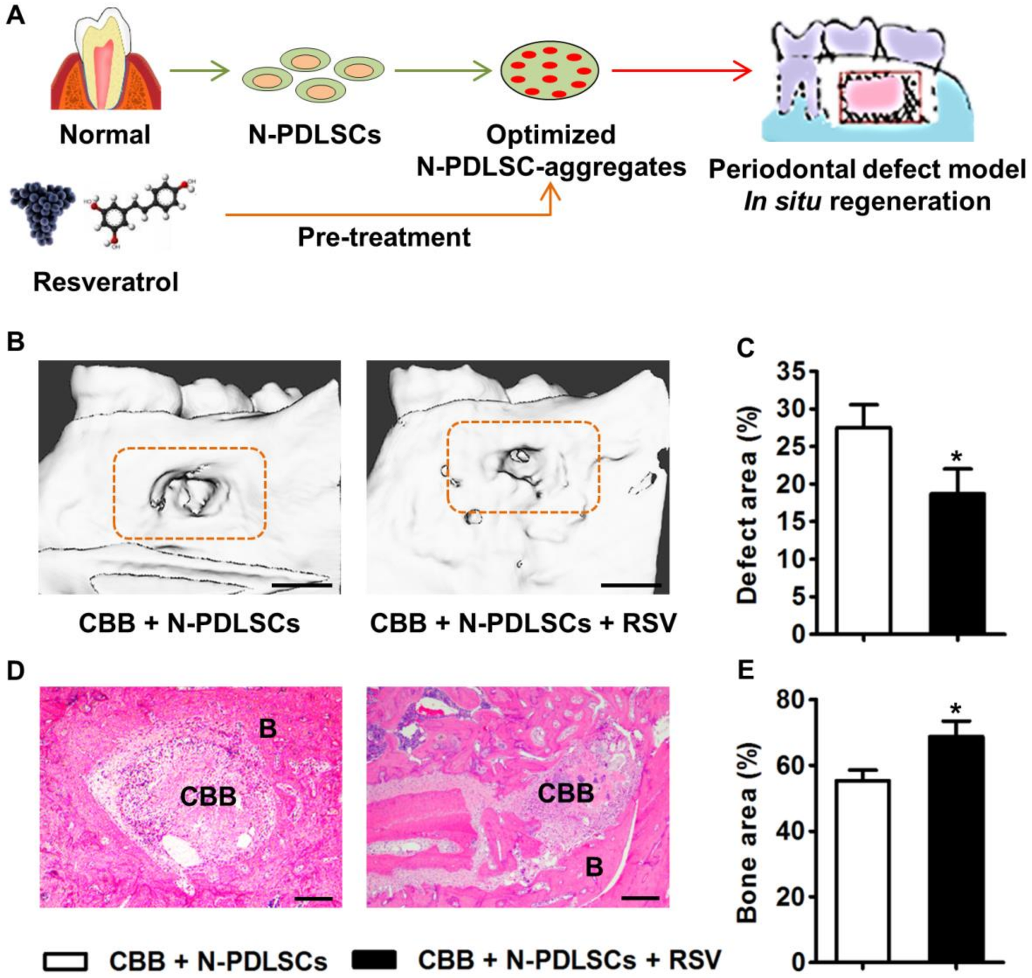

Figure S2. RSV treatment facilitated alveolar bone regeneration of N-PDLSC-aggregates in a rat periodontal defect model. (A) A schema illustrating the experimental design. (B, C) Representative micro-CT figures of periodontal defects after N-PDLSC-aggregate-based regeneration (B) and statistical analysis of bone volume per tissue volume (BV/TV) (C). The orange brackets indicate the original defects that were selected as region of interests in statistical analysis. (D, E) Histological analysis of periodontal bone regeneration (denoted by B) by N-PDLSC-aggregates by HE staining and the statistical analysis of regenerated bone area (E). RSV was applied at 10 nM throughout the *in vitro* treatments. Calcined bovine bone (CBB) was used as the scaffold. n = 4 per group. Data represents mean ± SD. *P < 0.05.
